# Early Use of Small Bowel Follow Through Reduces Stay and Cost in Small Bowel Obstructions

**DOI:** 10.7759/cureus.15023

**Published:** 2021-05-14

**Authors:** Mohammad Ali, Daniel R Slack, Richard Feinn, Scott Kurtzman, Zhongqiu J Zhang

**Affiliations:** 1 General Surgery, Waterbury Hospital, Waterbury, USA; 2 Surgery, Frank H. Netter MD School of Medicine at Quinnipiac University, Hamden, USA; 3 Statistics, Frank H. Netter MD School of Medicine at Quinnipiac University, Hamden, USA; 4 Surgery, Waterbury Hospital, Waterbury, USA; 5 Surgery, University of Connecticut School of Medicine, Farmington, USA; 6 General and Colorectal Surgery, Waterbury Hospital, Waterbury, USA

**Keywords:** sbft, sbo, los, gastrografin, costs, adhesions

## Abstract

Background

According to the Nationwide Inpatient Sample in 2011, nearly 1,500,000 admissions with over 300,000 laparotomies were performed for adhesion-related small bowel obstructions (SBOs). Small bowel follow through (SBFT) consists of serial X-rays with oral Gastrografin contrast that can diagnose obstructions requiring operative intervention. Furthermore, the contrast has a therapeutic osmotic effect which may promote transit and resolve an SBO. The aim of the study was to determine if early SBFT administration to patients with SBO decreases length of stay (LOS), hospital costs, and can identify patients who will fail non-operative management (NOM).

Methodology

This is a single institution retrospective study conducted from 2010 to 2019 with a total of 476 patients. We divided patients into three groups: SBFT within <24 hours of admission (n = 40), SBFT >24 hours after admission (n = 198), and did not receive SBFT (n = 238). We compared the overall LOS, hospital costs, and time from SBFT to the operating room using an analysis of variance.

Results

LOS significantly differed between groups with SBFT within ≤24 hours having an average LOS of 6.95 days compared to 10.65 days in the SBFT after >24 hours and 11.75 days in the no SBFT group (p = 0.005). Median time to the operating room in patients receiving SBFT was one day, which was significantly shorter than a median time of four days for no SBFT group (p = <0.05). Decreased LOS by 4.8 days equated to saving $8,657 per patient.

Conclusions

SBFT administered within 24 hours decreases LOS, overall costs, and time to operating room in patients who fail NOM.

## Introduction

Small bowel obstruction (SBO) is the most common cause of obstruction in adults secondary to adhesions due to a prior surgical procedure. When this condition occurs, there is an interruption in the normal transit of intestinal contents. Although 60 to 85% of SBOs secondary to adhesions resolve without any surgical intervention, it is challenging to predict which patients are at an increased risk of failing non-operative management (NOM) [1]. Small bowel compromise may result in ischemia, necrosis, or frank perforation which then result in the need for an emergent surgical intervention. SBO accounted for more than 300,000 hospitalizations and more than 846,000 days of inpatient care according to the Nationwide Inpatient Sample in 2011 [2]. Clinical signs for worsening bowel ischemia can be non-specific, though many patients demonstrate a combination of fever, leukocytosis, tachycardia, and worsening abdominal pain. Patients admitted with an SBO with four or more preoperative days prior to surgical intervention have an increased odds ratio of death compared to patients with fewer preoperative days [3]. These findings further support the need for an imaging study that will rapidly identify patients who fail NOM.

Oral water-soluble radiological contrast agents have been used in recent years to predict which patients will require surgery and which patients can be managed non-operatively. Small bowel follow through (SBFT) consists of serial abdominal X-rays with oral contrast that is diagnostic for obstructions and therapeutics with an osmotic effect to decrease time to resolution of an SBO [4] The aim of this study is to determine if SBFT administered to patients with moderate-to-severe SBO based on radiologic evidence decreases length of stay (LOS), hospital costs, and determine if it can rapidly identify patients who will fail NOM.

## Materials and methods

This is an Institutional Review Board-approved retrospective study at a community hospital, a single institution, including a total of 476 patients who had an SBO from 2010 to 2019. There were 238 patients with SBO who had received an SBFT and 238 patients within the same time frame who did not receive an SBFT. These 476 patients were organized into three groups: 40 patients received SBFT at <24 hours (group 1, n = 40), 198 patients received SBFT at >24 hours (group 2, n = 198), and 238 patients who did not receive SBFT (group 3, n = 238). A total of 35 out of 40 in group 1, 170 out of 198 in group 2, and 210 out of 238 in group 3 had a nasogastric tube (NGT). Patients who did not have an NGT were secondary to refusal during admission. The overall LOS, time from SBFT to discharge, and time from SBFT to the operating room in the setting of SBO failing NOM were compared. Inclusion criteria were SBO in patients secondary to adhesions from prior surgery, obstipation, and computed tomography (CT) scan confirming findings of moderate-to-high-grade obstruction. Exclusion criteria were patients who had SBO secondary to inflammatory bowel diseases, foreign body, or carcinomatosis; SBO without any prior surgical history; and hemodynamically unstable patient’s requiring immediate surgical intervention. The primary outcome was average LOS, and secondary outcomes included time from SBFT to operating room and time from SBFT to discharge along with hospital costs. The costs of an average stay along with costs of SBFT study were calculated for each individual patient and confirmed with billing department.

In the past, our hospital used mostly barium for SBFT studies; however, in 2016, Gastrografin was increasingly utilized. The quantity of either choice of administrated fluid was 300 cc; however, results of barium versus Gastrografin were not stratified.

An analysis of variance using the Brown-Forsythe robust test of means was used to compare group 1, group 2, and group 3 for continuous outcomes (LOS, time to surgery, and time to discharge), and the generalized linear model with logit link was used to compare groups on the dichotomous outcome having surgery. A significant group effect was followed-up with post-hoc Bonferroni pairwise comparisons to determine which groups differed.

## Results

A total of 476 patients were identified who were admitted for an SBO. There were 238 patients with SBO who had received an SBFT, and 238 patients within the same time frame who did not receive an SBFT. The patients were further divided into group 1 who received SBFT at <24 hours (n = 40), group 2 who underwent SBFT at >24 hours (n = 198), and group 3 who did not receive an SBFT (n = 236). A total of six (15%) patients in group 1 underwent operative intervention while 34 (85%) patients resolved with NOM, 50 (25%) patients in group 2 underwent operative intervention while 148 patients (75%) resolved with NOM. In group 3, 48 (20%) patients underwent operative intervention while 190 patients (80%) resolved with NOM.

Table 1 shows the descriptive statistics of the outcomes by SBFT grouping. LOS significantly differed between groups (p = 0.005) with group 1 patients having shorter stays than group 2 patients (p = 0.005) and group 3 patients (p < 0.001). Group 2 did not differ from Group 3 (p = 0.247). The average daily cost for an SBO patient was $1,803.50. Group 1 patients had an average LOS of 6.95 days compared to 11.75 days for group 3 patients. The shorter stay of 4.8 days equated to a saving of $8,657 average daily hospital costs per patient.

There was a significant difference between SBFT groups in the proportion of patients who had surgery (p = 0.008). Group 1 patients did not differ from group 3 patients (p = 1.00) or group 2 patients (p = 0.135), but a higher percentage of group 2 patients had surgery compared to group 3 patients (p = 0.013). Median time to the operating room in group 1 and group 2 was one day, while median time for group 3 was four days. This shorter duration was statistically significant (p = <0.05). Use of either barium or Gastrografin for SBFT had similar results for SBO patients in this study.

For patients who received SBFT, there was no significant difference in time from SBFT to discharge (p = 0.597). The average number of days to discharge from SBFT for patients in group 1 was 5.28 versus 6.12 days for patients in group 2.

**Table 1:** Descriptive statistics of the outcomes by SBFT groups. Mean ± SD for outcomes by SBFT status. SBFT: small bowel follow through

Outcome	Group 1 (SBFT ≤24 hours)	Group 2 (SBFT >24 hours)	Group 3 (No SBFT)	P-Value
Length of stay	6.95 ± 9.95	10.65 ± 10.06	11.75 ± 3.06	0.005
Surgery (%)	14.0%	26.3%	15.6%	0.008

## Discussion

The prevalence of SBOs due to adhesions in the United States is staggering with a huge impact on hospital resources, hospital costs, and patient quality of life. Patients who present with obstipation and CT evidence of a moderate or high-grade obstruction typically require hospital admission with bowel rest, intravenous fluids, pain, and nausea control. Though majority of these adhesion-related SBOs resolve on their own with NOM, the hospital LOS and costs are significant. As of yet, there is no established treatment to help these SBOs resolve. Adjuncts such as chewing gum and ambulation have long been thought to encourage return of bowel function; however, this has not been conclusively proven [5]. Our study of early SBFT use in SBO secondary to adhesions suggests an intervention that may help patients recover quicker.

SBFT has long been used as a diagnostic tool to discover small bowel obstructions, masses, anatomical abnormalities, and other small bowel pathology. While some studies have shown that Gastrografin/water-soluble contrast provides a therapeutic effect, no studies have evaluated the timing to administer SBFT for both diagnostic and therapeutic purpose [6].

Our retrospective study of 476 patients demonstrated a significantly decreased LOS as well as hospital cost when SBFT was administered within 24 hours. In addition, our results did not show a significant decrease in LOS in the group that received SBFT after 24 hours compared to the no SBFT group. This suggests that the true therapeutic benefit from SBFT is most effective when administered early in the patient’s treatment.

Our data also showed patients in group 2 were more likely to have surgery performed for their obstruction compared to both group 1 and group 3. This is more likely due to selection bias rather than the suggestion that late SBFT increases the likelihood of operation. Time to surgery was also noted to be longer in group 3 compared to group 2, suggesting that SBFT can aid in rapid decision-making and identify patients who will not improve with NOM [3,7].

Interestingly, we found that the length of time to discharge once SBFT was done was not significantly different between group 1 and group 2. This suggests that the therapeutic effect of SBFT was similar in both groups, and the difference in LOS was more dependent on how early it is administered. Average hospital costs were also seen to be significantly lower in the early SBFT, as would be expected given the decreased LOS. Thus, the included cost of an SBFT for a patient is much more offset by the amount saved on hospital days. Given the average decrease in LOS and average daily cost of a hospital stay, there is an average daily saving of $1,803.50.

The limitations of this study include the retrospective nature of a non-randomized group of patients. Furthermore, the decision to proceed with an SBFT and to choose diluted Gastrografin over diluted barium was attending-specific mostly for patients who were not demonstrating a consistent return of bowel function. The number of patients in group 1 was smaller compared to group 2 or group 3; however, it is rare for a study on SBFT to include patients receiving contrast material within 24 hours of admission. Although the data of the diluted Gastrografin and diluted barium were not stratified, the results suggest that timing is of the utmost importance and that an SBFT study regardless of contrast material gives objective data on contrast that will either pass into the colon or demonstrate a high-grade obstruction prompting operative management.

## Conclusions

Gastrografin or barium SBFT administered within 24 hours decreases LOS and overall hospital costs by identifying patients who will require operative intervention and by decreasing time to resolution in patient’s who will successfully resolve with the osmotic effect of an SBFT study. Large-scale prospective or randomized controlled studies should be considered.
